# Why a Chloroplast Needs Its Own Genome Tethered to the Thylakoid Membrane—Co‐Location for Redox Regulation

**DOI:** 10.1002/bies.70170

**Published:** 2026-08-06

**Authors:** John F. Allen

**Affiliations:** ^1^ Research Department of Genetics Evolution and Environment, Darwin Building, University College London London UK

**Keywords:** CoRR hypothesis, co‐transcriptional translation, co‐translational assembly, mesosome, nucleoid, thylakoid membrane insertion

## Abstract

A chloroplast is a subcellular organelle of photosynthesis in plant and algal cells. A chloroplast's genome encodes proteins of the photosynthetic electron transport chain along with proteins required to express them. Chloroplast‐encoded photosynthetic proteins function in close contact with proteins whose precursors are encoded in the cell nucleus for cytosolic synthesis, import into the chloroplast, and subsequent processing. Protein complexes of photosynthetic electron transport thus contain polypeptides with one of two quite different sites of synthesis. Most chloroplast proteins are products of nuclear genes. Why not all? One proposal is that photosynthetic electron transport governs expression of genes for its own components: co‐location of chloroplast genes with their gene products allows redox regulation of gene expression in individual organelles. Recent advances indicate that redox regulation impacts all stages of chloroplast gene expression. Here I propose that components of this regulation physically tether chloroplast DNA to the thylakoid membrane.

## Graphical abstract

A schematic outline of chloroplast DNA tethered to two stacked thylakoids that contain the components of photosynthetic electron transport. Green shapes represent proteins encoded in chloroplast DNA; brown shapes represent proteins encoded in the cell nucleus.

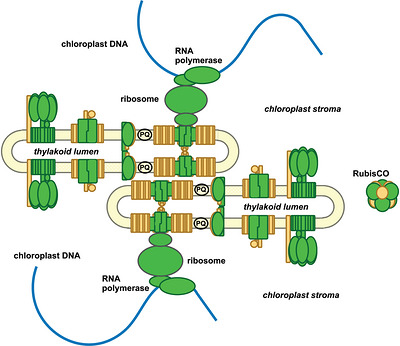

## 1 Introduction

Chloroplasts contain more than a thousand proteins [1], only a few percent of which are encoded by chloroplast DNA. The remaining chloroplast proteins are encoded by genes in the plant or algal cell nucleus, are synthesized on cytosolic ribosomes as precursor proteins, and are imported into the chloroplast stroma with the help of targeting signals that are recognized by the protein import machinery of the inner and outer chloroplast membranes [2, 3]. Chloroplast DNA can be transferred to nuclear genomes [4]. As a consequence, the genes for almost all chloroplast proteins have become firmly integrated into nuclear gene regulatory mechanisms and have evolved into permanent residents of nuclear chromosomes, with the original chloroplast‐encoded copy undergoing mutation and, eventually, loss [5]. But a small minority of genes for chloroplast proteins remain locked in place, unable to integrate into the nuclear genome. Different plant and algal lineages have converged independently upon the same set of chloroplast‐encoded proteins over the 1.5 billion years since the cyanobacterial origin of plastids [6]. What do these proteins have in common that has prohibited the successful relocation of their genes to the nucleus?

There are several proposals for the nature of selective pressures that maintain genes in organelles (reviewed in [7]). Most proposals focus on the physico‐chemical properties of individual proteins, such as hydrophobicity or importability. In contrast, one hypothesis, named Co‐location for Redox Regulation, abbreviated CoRR, focuses on the physiological properties of individual organelles—specifically, the biochemical activity of the photosynthetic electron transport chains within which most chloroplast‐encoded proteins function.

### 1.1 What's the Difference?

**FIGURE 1:**
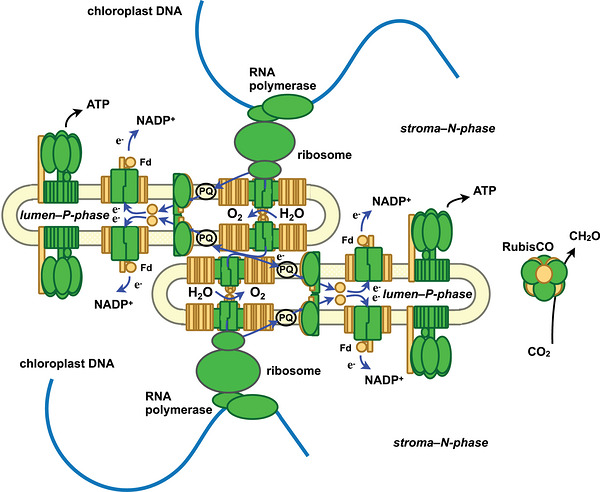
A schematic outline of chloroplast DNA tethered to two stacked thylakoids. Green shapes represent proteins encoded in chloroplast DNA; brown shapes represent proteins encoded in the cell nucleus. Blue arrows represent the path of photosynthetic electron transport. PQ, plastoquinone; Fd, ferredoxin. PQ and Fd are electron carriers whose redox state acts as a primary signal in regulation of photosynthesis [84].

A simple way to introduce and illustrate the difference between CoRR and other hypotheses for chloroplast gene retention is to start with the observation that chloroplasts encode genes for components of all major complexes of the photosynthetic electron transport chain (Figure 1): Photosystem II (PS II); the cytochrome *b*_6_ *f* complex; photosystem I (PS I); and RubisCO (the key enzyme of the Calvin cycle, the plant's proximal electron sink); plus the proton motive ATP synthase that is powered by the chain of electron transport from H_2_O to CO_2_. The flow of electrons through that chain needs to be regulated in order to remain balanced. In noncyclic electron transport and photophosphorylation, the rate at which electrons leave water at photosystem II must equal the rate at which electrons exit photosystem I to reach ferredoxin and NADP^+^ for CO_2_ (and thioredoxin) reduction. If there is any disruption or imbalance in the flow of electrons, components of the transport chain become oxidatively damaged, or worse, over‐reduced, enabling non‐enzymatic transfer of single electrons to O_2_, generating reactive oxygen species (ROS), oxidative damage and oxidative stress in the cell [8]; the starting point of a cascade that can quickly lead to cell death. The chloroplast needs to maintain electron flow in perfect redox balance.

As an illustration, consider a hypothetical plant cell with 100 chloroplasts, all lacking chloroplast DNA, and with all chloroplast proteins being encoded in the nucleus for import into the organelle. In one of these chloroplasts, a defect or physiological response arises that decreases the activity of PS I in the organelle. It then signals to the nucleus that more PS I is needed. The nucleus responds by increasing the amount of (nuclear‐encoded) PS I proteins. The nucleus knows which proteins to express, yes, but does not know which organelle needs them and has no mechanism to target them specifically to the chloroplast that sent the signal. The PS I deficit in our defective chloroplast can be remedied by a rapid supply of PS I precursors to the cytosol, but thereby giving the other 99 plastids too much PS I. The 99 that were fine at the outset now need more PS II and *b_6_f* to compensate, which they signal to the nucleus, which responds accordingly until some plastids send the signal “stop, more PS I, less *b_6_f*” etc. Signaling and gene regulation go haywire. The central gene regulatory machinery in the nucleus and cytosol cannot provide the stoichiometric amounts of proteins required to remedy redox imbalance within individual chloroplasts, each requiring precisely balanced electrical current through the two photosystems. The individual organelle of a plant cell needs to be able to regulate its own redox balance, like any free‐living prokaryote does [9], but how?

If, on the other hand, the chloroplasts in our hypothetical cell encode the standard set of chloroplast‐DNA‐encoded proteins, each organelle can individually respond to its own, individual need to maintain redox balance, under three conditions: (1) each chloroplast has the machinery required to express genes (DNA, RNA polymerases, ribosomes) in the organelle, (2) each chloroplast encodes components of each complex in the electron transport chain, and (3) each chloroplast has the means to sense the redox state of its own photosynthetic electron transport chain. If these conditions are met, then a defective chloroplast in need, for example, of more PS I can synthesize more PS I to restore *its own* redox balance, without disrupting the redox balance of the other 99 plastids in the cell, which can, in turn, respond to the status of their own redox state as needed. Of course, there are unicellular algae with only a single chloroplast per cell. Without CoRR, one‐nucleus‐one‐plastid might be expected to result in complete nuclear integration of chloroplast genes. There seems to be no example of this pattern of gene distribution.

The essence of the CoRR hypothesis is that chloroplasts (and mitochondria) need to be able to monitor and regulate their individual redox state by virtue of having genes for key components of the electron transport chain *Co*‐localized for *R*edox *R*egulation [10]. This strong selective pressure maintains genes for components of electron transport chains in organelles over evolutionary time, in addition to maintaining the organelle‐encoded machinery needed to express them. CoRR proposes that genes for a functional class of proteins—components of electron transport chains—are maintained in organelles by selection for optimally regulated electron flow. This phenomenon allows each individual organelle to maintain efficiency while avoiding oxidative stress caused by redox imbalance. CoRR's predictions are consistent with experimental results obtained from studies of photosynthetic gene expression, as described below.

### 1.2 Redox Control of Complete Protein Synthesis in Isolated Chloroplasts

Chloroplasts exhibit the property of regulated gene expression. Chloroplast gene expression is not constant across the light‐dark diel cycle, nor does it undergo random fluctuation. It is impacted by light, and light has direct and immediate effects on the redox state of photosynthetic electron carriers. Several independent lines of experimental evidence support the idea that chloroplast gene expression is under redox regulatory control [10].

The chloroplast contains a complete genetic system, from DNA replication and transcription to protein synthesis and assembly [11]. One compelling line of evidence for redox control of chloroplast gene regulation is direct measurement of total chloroplast protein synthesis by incorporation of ^35^S‐methionine into polypeptides synthesized within isolated pea chloroplasts [12]. The pattern of polypeptides newly synthesized in both thylakoids and stroma is unique to the redox conditions imposed during the incubation period in vitro. These conditions include the presence or absence of specific chemical oxidants and reductants. Furthermore, distinct patterns of polypeptide labelling are obtained in light and darkness, with the light‐dependent pattern being influenced by the presence or absence of the site‐specific inhibitors of photosynthetic electron transport DCMU and DBMIB. Because the redox sensing and gene expression take place in the absence of nucleus and cytosol, it is concluded that expression of chloroplast genes depends on the redox state of components within the chloroplast itself. The effects of electron transport inhibitors demonstrate that light itself is not the stimulus impacting gene expression; it is the redox state of one or more components of chloroplast electron transport.

### 1.3 Redox Control of Chloroplast Transcription

Chloroplasts evolved from cyanobacteria. Redox control of transcription for components of electron transport in prokaryotes entails the operation of either a two‐component redox regulatory system or a single‐component redox activator or repressor [13]. In a series of experiments with chloroplasts isolated from mustard seedlings, synthesis of specific mRNAs took place in light of spectral composition favoring either photosystem I or photosystem II [14, 15]. Plastoquinone is an electron carrier located between photosystem II and photosystem I; its redox state is determined directly by the two photosystems’ relative rates of electron transfer. Experiments with both mustard and pea chloroplasts indicate that the redox state of plastoquinone exerts control over transcription of genes for reaction centre apoproteins [14, 15, 16]. Light that is absorbed predominantly by photosystem II reduces the plastoquinone pool. Reduced plastoquinone acts as a signal that photosystem I is rate‐limiting. Accordingly, absorbed light energy becomes re‐distributed in favor of photosystem I, and genes for photosystem I reaction center proteins are switched on. Conversely, when spectral composition favors photosystem I, plastoquinone is oxidized, absorbed light energy becomes re‐distributed in favor of photosystem II, and photosystem I reaction center genes are repressed. These effects correct for imbalance in the stoichiometry of photosystem I and photosystem II [14, 17]. Initiation of transcription of genes for chloroplast proteins requires characteristically prokaryotic RNA polymerase subunits known as sigma factors [18]. A number of factors exert regulatory control over photosynthesis and chloroplast function generally. These include light quality, light intensity, nutrient status, and plastid developmental stage [19]. Nevertheless, transcriptional initiation is proposed as a key control point for chloroplast gene expression [20, 21, 22] and for assembly of protein complexes containing multiple subunits. This control may involve a typically bacterial two‐component system [23], and is likely to have been a conserved requirement in the ancestral cyanobacterium, in endosymbiosis, and in chloroplasts. In line with this view, a number of redox enzymes coupled to the chloroplast transcriptional apparatus [24, 25] become tethered to the thylakoid membrane when transcription is active [26].

### 1.4 Translation and Protein Folding in Chloroplasts

In addition to transcriptional regulation, regulation of gene expression also takes place at the level of translation, and several factors influence translation of chloroplast mRNA [27]. Folding of polypeptides generally occurs during synthesis [28], not after it, and begins within the exit tunnel of ribosomes [29, 30]. This folding is mediated by a chain of molecular chaperones [31] without which non‐functional protein‐protein interactions take place [32]. Early, guided protein folding may be of special importance for intrinsic membrane proteins whose hydrophobic amino acid side chains will otherwise promote protein aggregation. Furthermore, if the functional partner of the nascent membrane protein is itself a component of a pre‐existing membrane protein complex, then the ribosome engaged in translation becomes bound to the thylakoid membrane [26]. Cryo‐electron tomography of isolated, intact spinach chloroplasts reveals particles identified as ribosomes within 15 nm of the stroma‐exposed “top” surface of thylakoid stacks [33]. In *Chlamydomonas*, chloroplast ribosomes are seen in the stroma, free, and in association with polysomes. About 31% of these chloroplast ribosomes, or “chlororibosomes”, are bound to thylakoid membranes where they are distributed along the stromal surface of thylakoid stacks [34]. Chlororibosomes actively translate both soluble, stromal proteins and membrane proteins of the photosynthetic machinery. CoRR posits that regulated gene expression in chloroplasts is the selective force behind the retention of genes in chloroplast DNA.

### 1.5 Membrane Insertion During Assembly of Photosynthetic Protein Complexes

As outlined above, electron flow through photosystem II and photosystem I needs to be stoichiometrically balanced. Chloroplast gene regulation helps to ensure physiological electron flow in the photosynthetic membrane. The D1 protein, the *psbA* gene product, of the reaction center of photosystem II becomes rapidly broken down and re‐synthesised during a photosystem II repair cycle [35, 36, 37]. This cycle requires insertion of the membrane‐intrinsic and hydrophobic D1 protein together with a post‐translational cleavage of an amino‐terminal segment of its precursor [38]. In cyanobacteria, the D1 precursor accumulates at thylakoid convergence zones [39]. Without exception, D1 in nature is never encoded in the cell nucleus, and is always encoded, as *psbA*, in the chloroplast genome. A nuclearly‐encoded D1 has been engineered to provide for allotopic expression of *psbA* complementary DNA driven by a heat‐responsive promoter in the nuclear genome [40].

For photosystem I, in addition to the hydrophobic nature of membrane‐intrinsic reaction center proteins, chloroplasts of both *Chlamydomonas* and land plants contain nuclearly encoded thylakoid‐bound photosystem I assembly factors [41]. Photosystem I assembly begins with the co‐translational insertion of the proteins psaA and psaB into the thylakoid membrane to form the heterodimer of psaA and psaB in the reaction center of photosystem [41]. While nuclearly encoded proteins are also involved in photosystem biogenesis, the encoding of photosystem components in chloroplast DNA gives individual plastids a voice in the decision‐making process of which photosystem is in need of replenishment.

### 1.6 Tethering the Genome to the Thylakoid: From DNA Transcription to Assembly of Photosynthetic Membrane Protein Complexes

Co‐transcriptional translation is seen in prokaryotic systems [42, 43]. I suggest that the chloroplast, derived from a cyanobacterium [44, 45], is no exception. If folding and insertion of nascent polypeptides accompany the earliest stages of translation [46], and if translation occurs on ribosomes connected physically to the plastid‐encoded RNA polymerase [24], then a structural connection must exist between the thylakoid and chloroplast DNA. Co‐transcriptional translation and co‐translational assembly together indicate tethering between the chloroplast genome and the photosynthetic apparatus. This tethering is outlined schematically in Figure 1.

### 1.7 A Chloroplast Mesosome?

A mesosome is a point of attachment of DNA to a cell membrane in prokaryotes [47, 48]. Mesosomes were first observed by transmission electron microscopy of heavy‐metal‐stained cells, raising the possibility that they are artifacts of sample preparation [49]. In early studies, there was no obvious reason for a physical connection between a biological membrane and DNA, and several suggestions were made regarding their possible significance [48, 50]. These suggestions tended to focus on cell division [51, 52]. Noting a possible connection of the mesosome with redox reactions of the bacterial respiratory chain, Reusch and Burger wrote as follows: “Earlier expectations that the mesosome might represent the mitochondrion of the bacterial cell are not likely to be fulfilled. It would be of interest to study any possible relationship between the photosynthetic apparatus and mesosomes in photosynthetic bacteria” [47].

### 1.8 The Chloroplast Nucleoid

A nucleoid was originally thought to be a bacterial equivalent of the eukaryotic cell nucleus, also with a focus on its possible role in cell division [53]. Today the idea of a chloroplast mesosome seems to have fallen out of favor, while there are active studies and a detailed composition [54, 55] of what is agreed to be a chloroplast nucleoid: “In bacteria the genome is organized as a huge protein/DNA complex that is not separated from the cytosol by a nuclear double membrane as in eukaryotes. It is thus accessible to the transcription and translation machineries at the same time. The plastome is organized in a similar manner…” [54]. Chloroplast transcription can be studied in a transcriptionally active chromosome, or TAC, that contains DNA, RNA, and around 35 proteins; at least five of which are predicted to be membrane‐bound [54]. Protein and RNA structural features are seen in a model derived from cryo‐electron microscopy of the complete spinach chloroplast ribosome at 3.0 Å resolution [56].

### 1.9 Chloroplast mRNA Targeting

Cyanobacterial mRNAs that encode core photosynthetic membrane proteins are themselves attached to the thylakoid by means of membrane‐associated RNA‐binding proteins [57]. Co‐translational folding and assembly indicate co‐translational insertion of nascent polypeptide chains, or “transertion”: “a system for membrane protein production in which transcription of an mRNA and co‐translational insertion of its translation product occur simultaneously. It requires the genomic locus encoding the protein to be in close proximity to the target membrane” [57]. It is proposed [57] that transertion was maintained throughout the endosymbiotic incorporation of a cyanobacterium into the cytoplasm of a eukaryote, and that this process required the maintenance of an internal genome to supply the mRNAs required. Co‐transcriptional translation together with co‐translational insertion of precursor polypeptides requires tethering between the chloroplast genome and the photosynthetic apparatus, as outlined schematically in Figure 1. Transertion [57] provides direct evidence of physical interaction between regulated gene expression and the regulated function of the photosynthetic electron transport chain, the salient element of the CoRR hypothesis.

### 1.10 Integrated Redox Control of Multiple Stages of Chloroplast Gene Expression

The CoRR hypothesis can be described as the need for DNA internal to the chloroplast in order to permit redox regulatory control of transcription [7, 10, 58, 59, 60]. There are independent lines of evidence indicating the existence of such control [14, 15, 21, 61]. Indeed, transcriptional initiation may be a primary control point in chloroplast gene expression [22]. Nevertheless, there are clear indications that every stage of chloroplast gene expression is under redox control, and that none, on its own, is sufficient to explain integrated photosynthetic redox signaling [62]. Post‐transcriptional regulation of plastid gene expression may occur by means of RNA processing that is under the control of nuclear genes [63, 64]. Ribosomal DNA footprinting reveals multiple epistatic effects of mutations in chloroplast DNA [65]. *psbA* translation is light‐regulated by means of a factor or factors involved in the photosystem II repair cycle [66, 67].

Other theories for organelle genome retention emphasize the need for light‐regulated gene expression, including Control by Epistasy of Synthesis (CES) [68, 69, 70] in which synthesis of chloroplast‐encoded photosynthetic subunits is regulated by the assembly state of the complexes in which they function. In angiosperms, results from ribosome profiling and RNAseq suggest that *psbA* is the only gene in a mature chloroplast whose expression is regulated by light, with this regulation occurring at the level of translation [71]. Given an apparent switch between transcription and translation during development [19], this conclusion may be consistent with the observed control of *psbA* transcription in seedlings of mustard [15] and pea [16]. In plastids of dinoflagellates, there is also CO‐location for Control of Assembly (COCOA) [72], a process that clearly depends on gene retention. These proposals are not at all inconsistent with the CoRR hypothesis. On the contrary, they may be seen as special cases of CoRR's central postulate: the non‐negotiable evolutionary requirement for regulated function of bioenergetic membranes as the selective pressure that maintains genes (hence DNA) in organelles. In particular, the redox state of the thylakoid plastoquinone pool may be decisive for numerous regulatory responses to environmental stress [73].

### 1.11 Inference From Comparison of Organellar Genomes

Evolutionary retention of genes in organelles is correlated with hydrophobicity of protein products of organellar gene expression [74]; with GC content; and with “the centrality of a protein product in its functional complex” [75]. Hydrophobicity alone has been proposed as the decisive factor for gene retention, particularly for mitochondria [76], with subsequent enquiries then focusing on possible barriers to import of protein precursors from the cytosol [77]. In such studies, there seems to be a tacit assumption that endosymbiotic gene transfer [78]—migration of genes from an endosymbiont to the eukaryotic cell nucleus [79, 80]—is inevitable, even if slow [81], and thus reasons must be found for barriers to this migration in the case of specific genes retained by an organelle. This and other proposals for retention of organellar genomes have been reviewed [7].  Both chloroplasts and mitochondria have retained genes for proteins of their respective bioenergetic membranes [6]. This remarkable convergence is, I propose, the result of the same selective force—the need for balanced electron flow—acting upon independently evolving units of physiological function: the photosynthetic and respiratory electron transport chains.

## 2 Conclusion

There is no structural or physicochemical feature that is common to all chloroplast‐encoded gene products. Instead, conserved patterns of gene location are consistent with photosynthetic redox chemistry exerting gene regulatory control over its own rate‐limiting steps [82]. Chloroplast DNA carries genes whose expression is placed under this control. The CoRR hypothesis proposes that gene location in chloroplasts and mitochondria is itself the result of natural selection, and predicts that genes will never disappear completely from photosynthetic organelles. This view seems to apply most obviously in the case of chloroplasts [83], where photosynthetic redox control of chloroplast gene expression [59] is clearly established [10]. This redox regulatory control may be exerted by means of physical tethering of chloroplast DNA to the chloroplast thylakoid membrane.

## Author Contribution

John F. Allen wrote this paper, and drew the figure.

## Conflicts of Interest

The authors declare no conflicts of interest.

## Data Availability

The authors have nothing to report.
